# Factors Influencing the Likelihood of Alcohol and Tobacco Use in Adolescent Athletes: Type of Sport, Age, and Action Tendencies in Sport

**DOI:** 10.3390/children10071248

**Published:** 2023-07-20

**Authors:** Alejandro Carriedo, José Antonio Cecchini, Antonio Méndez-Giménez

**Affiliations:** Department of Education Sciences, Faculty of Teacher Training and Education, University of Oviedo, 33003 Oviedo, Spain; carriedoalejandro@uniovi.es (A.C.); mendezantonio@uniovi.es (A.M.-G.)

**Keywords:** drugs, lifestyle, aggressiveness, submissiveness, moral reasoning

## Abstract

This article proposes a new approach to understand substance use among adolescent athletes. Thus, this article describes an investigation of the likelihood of alcohol and tobacco use in adolescent athletes considering the type of sport, age, gender, and their action tendencies. A total of 552 athletes with an age range of 12–16 years were engaged in medium–high-contact sports (*n* = 291) and non-contact sports (*n* = 261). They completed the Children’s Action Tendency Scale, the Sport Children’s Action Tendency Scale, and the Personal and Social Responsibility Questionnaire. The results showed that 16-year-old athletes were the most likely to drink alcohol and to smoke tobacco (*p* < 0.001). Likewise, it was found that practicing a medium–high-contact sport was a risk factor for alcohol and tobacco use (*p* < 0.01). The more aggressive athletes were more likely to have smoked tobacco during the last month (*p* = 0.019) and also to drink more alcohol that the less aggressive ones (*p* < 0.001). Finally, the athletes who showed more submissiveness were less likely to have drunk alcohol in the last year and the last month (*p* < 0.001). These findings show the predictive value of new factors associated with alcohol and tobacco use among adolescent athletes. Preventive measures should be targeted, since a new link between the action tendencies in sport and substance use has been found.

## 1. Introduction

Sport is a very important leisure activity among adolescents. The latest survey of sporting habits in Spain registered that 81.2% of young people aged between 15 and 19 years old were involved in at least one sporting activity and that the percentage of young people with at least one sports license was around 20% [1]. However, a recent study revealed that 76.6% of Spanish adolescents did not meet the recommendations for aerobic exercise in 2016, and similar results were observed globally: 81% of students aged 11–17 years were insufficiently active [2].

Participation in organized sports in adolescence is associated with higher physical activity [3]. Thus, adolescents may accumulate a minimum of moderate–vigorous physical activity per week, which is associated with multiple health benefits. For instance, physical activity and practicing a sport in youth may include better overall mental health [4] and better cardiometabolic health in youth [5]. Additionally, positive emotional [6] and social benefits have been observed, such as improved social identity and social adjustment in adolescents [7].

Sports are commonly included in school curricula and have also been considered a tool to prevent problem behavior in adolescence [8]. Physical activity and exercise have been suggested as a potential therapeutic intervention against the use of tobacco, alcohol, and illicit drugs [9,10]. However, the literature is inconsistent regarding sports participation as a protective or a risk factor for drug use [11,12]. While some studies have reported negative associations between sports practice and alcohol [13] and tobacco use [9,14], others have claimed that playing high school sports seems not to be a protective factor for substance use in youth [8,15]. For instance, although negative associations between the initial level of participation in organized sports and alcohol intoxication have been found [16], Zubak et al. [12] observed that drug initiation was more probable in adolescents who practice sports for a prolonged period of time.

Adolescents who played sports also exhibited a higher frequency of alcohol use than their peers who did not play sports [15]. However, according to Zubak et al. [12], quitting sports is considered a risk factor for the earlier initiation of illicit drug misuse (i.e., before 16 years of age). On the other hand, it should be noted that not all sports/physical activities may have a positive relationship with substance use among adolescents [17], suggesting different patterns of drugs use among adolescents regarding the type of sports practiced.

Thus, it has been detected that sports participation in adolescence and participation in team sports are positively associated with alcohol consumption during late adolescence, whereas participation in team sports and endurance sports may reduce later use of tobacco [16]. This is very worrisome, because adolescent alcohol exposure is a risk for prolonged alcohol-related problems [18] and is associated with mental health problems and other functional deficits such as verbal learning, attention, and visuospatial and memory tasks [19].

Antisocial behavior is related to the consumption of tobacco and alcohol by adolescents [20]. In the sport context, the term antisocial behavior refers to actions intended to harm or disadvantage another athlete [21]. Some action responses related to antisocial behavior in sports may include aggressiveness towards opponents and even teammates [22]. It has been proposed that children’s moral reasoning may predict assertiveness, aggressiveness, and submissiveness in conflict situations both in sport and daily life [23]. These action tendencies include three types of responses to situations that could occur in different social conflicts [24]. While assertiveness means defending one’s rights while respecting the rights of others, aggressiveness and submissiveness mean defending one’s right by violating others’ rights, and subordinating ones’ rights to those of others, respectively [25].

In this regard, aggressiveness has been associated with alcohol [26] and tobacco consumption [27]. With the aim of providing further insight into the associations between sports participation and alcohol and tobacco use in adolescents, new questions are proposed. For instance, could the action tendencies in life and/or in sports and the responsibility of adolescent athletes be related to their alcohol and tobacco use? Although practicing a sport could protect against the use of tobacco, alcohol, and illicit drugs [9,10], it is important to obtain a better understanding of these relationships. In this regard, the study of variables such as the type of sport practiced, the adolescent athletes’ action tendencies, and their responsibility in sports could provide new information to detect the early risk of alcohol and tobacco use among adolescents.

No studies examining the relationship between substance use among adolescent athletes and their action tendencies and personal and social responsibility in sports were found in the scientific literature. Based on the aforementioned background, this study aimed to investigate the likelihood of alcohol consumption and tobacco use in adolescent athletes according to the type of sport (low-, medium-, or high-contact sport), age, gender, their action tendencies in daily life and in sport (assertiveness, submissiveness, aggressiveness), and their responsibilities (personal and social).

## 2. Materials and Methods

### 2.1. Participants and Procedure

The sample of this correlational study comprised 552 adolescent athletes from a city located in northern Spain (216 males and 336 females), aged 12–16 years (*M_age_* = 13.52 years, *SD* = 1.46 years). A total of 291 participated in medium- and high-contact sports (basketball (*n* = 159), rugby (*n* = 102)), whereas the remaining 261 participants participated in non-contact sports (badminton (*n* = 90), speed skating (*n* = 39), synchronized swimming (*n* = 75), and triathlon (*n* = 87). All were Caucasian with no health issues. The convenience sample was taken from different athletes that belonged to sports clubs in the municipality of Oviedo, which carry out activities that favor the promotion and dissemination of sports.

The inclusion criteria were age between 12 and 16 years old and they must be enrolled in any of the sports clubs that participate in the competitions organized by the city council of Oviedo. The participants also had to declare that they were in good health and to confirm that they participate regularly in the training and events organized by the sports club.

This study was developed through a cooperation agreement between the local community Plan on Drugs (city council from Oviedo) and the University of Oviedo. First, permission to conduct the study was obtained from the researchers’ State Ethics Research Committee. Then, the research team met with the presidents, coaches, and athletes from different sport teams that were selected by convenience sampling to obtain parental signed informed consent. The participating athletes filled out a self-report questionnaire that measured their action tendencies (assertiveness, submissiveness, aggressiveness) in sport and other daily contexts, and their personal and social responsibilities during the training sessions. The whole procedure was conducted by a researcher who ensured that no other person was present at the moment of collecting data and also informed the participants that their responses would be anonymous and they could withdraw from the study at any time.

### 2.2. Instruments and Measures

*The Children’s Action Tendency Scale (CATS)* assesses the moral reasoning regarding assertive, aggressive, and submissive behaviors in young people [28]. The athletes answered questions regarding daily life situations that can lead to frustration, provocation, and conflict (e.g., “You are making a puzzle with your friends. You have tried to do it right, but you cannot avoid making some mistakes. Your friends begin to tease you and call you names. What would you do”). Each scenario was followed by three alternative options: (a) assertiveness: “I would tell them to calm down, because they would not enjoy being teased either”; (b) aggressiveness: “I would hit the one that is teasing me more”; and (c) submissiveness: “I would stop playing and I would go home”.

The options were presented in a pairwise fashion and the participants had to choose one of each possibility: assertiveness vs. submissiveness, aggressiveness vs. submissiveness, and aggressiveness vs. assertiveness. The short version of six questions and 18 responses translated into Spanish [29] was used. Thus, the total scores for each subscale ranged from 0 to 12. In the present study, the Kuder–Richardson formula (K-R-20) for dichotomic answers was used to assess the internal consistency of each subscale. The scores obtained were assertiveness = 0.58, aggressiveness = 0.77, and submissiveness = 0.60, which were similar to Bredemeier’s scores (1994; assertiveness = 0.54, aggressiveness = 0.80, and submissiveness = 0.58). The K-R-20 values should be interpreted as estimates with a paired comparison procedure. For instance, an assertive boy may frequently select assertive alternatives. However, he is forced to choose between submissive and aggressive alternatives in one third of the cases. Nevertheless, it can be expected that he selects assertive alternatives where possible and, therefore, to have a low selection rate for the submissive and aggressive alternatives.

The *Sport Children’s Action Tendency Scale (SCATS*) measures the same variables as the preceding instrument, but in the sport context [23]. Hence, it follows the same structures. The adolescent athletes answered questions regarding situations that can lead to assertiveness, aggressiveness, or submissiveness in sport (e.g., “Your team is in a basketball tournament. A player from the other team pushes one of your teammates to the floor, hurting his/her knee and forcing him/her to leave the court. What would you do?”). Each scenario is followed by three alternative options: aggressiveness: “I would get angry and try to push that player to the floor”; assertiveness: “I would tell that player that he/she should not play like that”; and submissiveness: “I would remain far from that player to avoid being hurt”.

The options were also presented in a pairwise fashion such as in the preceding instrument. The participants had to choose one of each. The total scores for each subscale ranged from 0 to 20. The internal consistency of each subscale was also assessed using the Kuder–Richardson formula (K-R-20) for dichotomic answers. The scores obtained were (the scores from the original study are shown in parentheses): assertiveness = 0.73 (0.68), aggressiveness = 0.84 (0.85), and submissiveness = 0.69 (0.66). These scores should be interpreted as estimates within a paired procedure of comparison.

In terms of the *Personal and Social Responsibility Questionnaire* [30], the Spanish validated version [31] was used. This scale includes 14 items, distributed evenly between two factors: Social Responsibility (i.e., “I try hard”) and Personal Responsibility (i.e., “I respect others”). The participants responded to the stem: “In the training sessions…” using a Likert scale ranging from 1 (strongly disagree) to 5 (strongly agree). The Cronbach’s alphas obtained in the present study were 0.83 and 0.76.

*Substance use.* To obtain information about an adolescents’ alcohol and tobacco use, additional questions were included in the questionnaire: “How many times have you had an alcoholic beverage (more than a few sips)?”, and “How many times you smoked cigarettes?”. In both questions, the participants had to respond to the following questions: (a) throughout your life; (b) during the last 12 months; (c) during the last 30 days, using an ordinal scale of seven options (i.e., 0, 1–2, 3–5, 6–9, 10–19, 20–39, 40 or more). Similar questions have been included in prior research assessing substance use among adolescents [14,27,32].

### 2.3. Data Analysis

All data were analyzed using SPSS version 24.0 (IBM Co. Ltd, Chicago, IL, USA). Multivariate ordinal logistic regression analyses were executed for examining the associations between the multiple independent variables proposed (i.e., type of sport (contact vs. non-contact sport), age, gender, action tendencies (aggressiveness, assertiveness, submissiveness) in life and in sport, and personal and social responsibility in sport) and alcohol and tobacco use in adolescent athletes (a) throughout life; (b) during the last 12 months; and (c) during the last 30 days.

## 3. Results

### Ordinal Logistic Regression

The first multivariate ordinal logistic regression model analyzed the proposed influencing factors (i.e., type of sport, age, gender, action tendencies in life and in sport, and personal and social responsibility in sport) of smoking cigarettes throughout life. The results showed that the model fit was good, *χ*^2^(6) = 39.425, *p* = 0.000. Approximately 15% of the variance of smoking 0, 1–2, 3–5, 6–9, 10–19, 20–39, or more than 40 cigarettes throughout life can be explained by the factors included in the model (Nagelkerke *R*^2^ = 0.146). Table 1 shows an overview of the regression coefficients for each significant factor. As can be seen, 16-year-old athletes are more likely to report a higher rate of smoking cigarettes throughout life when compared to the remaining ages. In addition, athletes who participate in medium- or high-contact sports are also more likely to report the highest rates of smoking cigarettes compared to athletes who practice sports without physical contact with opponents.

The second multivariate ordinal logistic regression model analyzed the proposed influencing factors of smoking cigarettes during the last 12 months. The results showed that the model fit was good, *χ*^2^(5) = 44.59, *p* = 0.000. Approximately 19% of the variance of smoking 0, 1–2, 3–5, 6–9, 10–19, 20–39, or more than 40 cigarettes during the last year can be explained by the factors included in the model (Nagelkerke *R*^2^ = 0.186). Table 2 shows an overview of the regression coefficients for each significant factor. As can be seen, the results are similar to the previous model.

The third multivariate ordinal logistic regression model analyzed the proposed influencing factors of smoking cigarettes during the last 30 days. The results showed that the model fit was good, *χ*^2^(6) = 32.70, *p* = 0.000. Approximately 22% of the variance of smoking 0, 1–2, 3–5, 6–9, 10–19, 20–39, or more than 40 cigarettes during the last month can be explained by the factors included in the model (Nagelkerke *R*^2^ = 0.221). Table 3 shows an overview of the regression coefficients for each significant factor. The results are similar to those of the previous models. In addition, athletes who showed more aggressive tendencies in their sport also were more likely to report a higher rate of smoking cigarettes during the last 30 days.

The fourth multivariate ordinal logistic regression model analyzed the proposed influencing factors (i.e., type of sport, age, gender, action tendencies in life and in sport, and personal and social responsibility in sport) of drinking alcoholic beverages throughout life. The results showed that the model fit was good, *χ*^2^(6) = 78.68, *p* = 0.000. Approximately 23% of the variance of drinking 0, 1–2, 3–5, 6–9, 10–19, 20–39, or more than 40 alcoholic beverages throughout life can be explained by the factors included in the model (Nagelkerke *R*^2^ = 0.228). Table 4 shows an overview of the regression coefficients for each significant factor. As can be seen, 16-year-old athletes were more likely to report a higher rate of drinking alcoholic drinks throughout life when compared to athletes aged 12–14 years. Athletes who participated in medium- or high-contact sports were also more likely to report the highest rates of drinking alcohol compared to athletes who practiced sports without physical contact with opponents. Finally, athletes who showed more aggressive tendencies in their sport also were more likely to report a higher rate of drinking alcohol throughout life.

The fifth multivariate ordinal logistic regression model analyzed the proposed influencing factors of drinking alcoholic beverages during the last 12 months. The results showed that the model fit was good, *χ*^2^(5) = 88.15, *p* = 0.000. Approximately 26% of the variance of drinking 0, 1–2, 3–5, 6–9, 10–19, 20–39, or more than 40 alcoholic beverages during the last year can be explained by the factors included in the model (Nagelkerke *R*^2^ = 0.258). Table 5 shows an overview of the regression coefficients for each significant factor. As can be seen, 16-year-old athletes were also more likely to report a higher rate of drinking alcoholic drinks during the last year when compared to athletes aged 12–14 years. Moreover, athletes who participated in medium- or high-contact sports were also more likely to report the highest rates of drinking alcohol compared to athletes who practice sports without physical contact with opponents. Finally, athletes who showed more submissive tendencies in their sport were also more likely to report a lower rate of drinking alcohol during the last 12 months.

The last multivariate ordinal logistic regression model analyzed the proposed influencing factors of drinking alcoholic beverages during the last 30 days. The results showed that the model fit was good, *χ*^2^(5) = 56.04, *p* = 0.000. Approximately 18% of the variance of drinking 0, 1–2, 3–5, 6–9, 10–19, 20–39, or more than 40 alcoholic beverages during the last month can be explained by the factors included in the model (Nagelkerke *R*^2^ = 0.182). Table 6 shows an overview of the regression coefficients for each significant factor. Once again, 16-year-old athletes were more likely to report a higher rate of drinking alcoholic beverages during the last 30 days when compared to athletes aged 12–14 years. Likewise, athletes who showed more submissive tendencies in their sport were also more likely to report a lower rate of drinking alcoholic beverages during the last 30 days.

## 4. Discussion

This study aimed to investigate the likelihood of alcohol consumption and tobacco use in adolescent athletes according to the type of sport, age, gender, their action tendencies in daily life and in sport, and their responsibilities in sport. Adolescence is considered a significant period in the development of health risk behaviors, for instance, alcohol or tobacco consumption [33]. The results of this study suggest that adolescent athletes around 16 years old are more likely to smoke tobacco throughout life, during the last 12 months, and during the last 30 days than adolescent athletes aged under 16 years old. Likewise, athletes aged 16 years old are more likely to drink alcohol than athletes aged 12–14 years. Based on the results of previous studies, 14–16-year-old adolescents are at higher risk of developing the habit of smoking [34] and the co-occurrence of smoking and drinking [32,35]. A recent study conducted with adolescent female football players observed that few players used tobacco and alcohol, but drinking alcohol appeared to increase with age [36]. The results of the present work also highlight the problematic situation that 16-year-old athletes could be facing when it comes to alcohol and tobacco use.

However, although the alcohol consumption was higher among 16-year-old athletes, there were no significant differences between adolescents aged 15 and 16 years. This suggests, in contrast to the tobacco use, higher rates of early alcohol consumption in adolescent athletes. These results concur with the scientific literature related to substance use among adolescents. The typical age of initiation of alcohol consumption in Spain is 14 years for both sexes; 78% of adolescents between 15 and 16 years old have consumed alcohol at some time in their lives, and 47% in the last 30 days [37].

Most studies found that adolescent athletes smoke less often than other adolescents [16,38]. However, the association with alcohol consumption is less consistent. Thus, some studies observed that alcohol consumption was higher in adolescents involved in sports [39,40], whereas others indicated no association [11] or positive effects [13,41,42]. Various possible explanations have been proposed. On the one hand, sports participation is a social activity, and therefore it could increase the risk of alcohol and drug use [16]. A second alternative explanation is that sports participation can be very stressful for individuals who aspire to be professional athletes, and high-level athletes are constantly being tested and evaluated on their performance. Hence, alcohol might be introduced as a coping strategy for sport-related anxiety [38].

Watching televised sports events, during which commercials may appear for alcohol products, could be another factor [43]. On the other hand, sports participation may reduce the risk of substance use because of the reduced contact with older adolescents, which increases the risk of smoking and alcohol intoxication [44]. Moreover, other possibilities, such as the time required to practice a sport in a competitive way, adult supervision during the competitive season, or the inverse relationship that exists between physical fitness/performance and alcohol and tobacco use could theoretically explain a reduction of the risk of alcohol and tobacco use in adolescents [16]. In this regard, several studies have shown that higher levels of exercise were associated with lower levels of alcohol and cigarette use [9,45]. Thus, the exercising habits among adolescents could be a more consistent predictor of alcohol and tobacco consumption than participating in sports.

It should be noted that these relationships might be dependent on the sport [17,46], and the adolescent athletes’ action tendencies in sport could also be related. Although further research is necessary to more clearly describe the link between sport participation and substance use, the overall results of this study suggest that practicing a medium- or high-contact sport and showing more aggressive tendencies in the sport context might increase the likelihood of alcohol and tobacco use during adolescence, especially at the age of 16 years old. These results suggest that the type of sport practiced could be a predictor of alcohol and tobacco use among adolescents.

This possibility was previously evaluated by Moore and Chudle [17], who proposed that the substance use among adolescents would be different according to the participation in specific school-sponsored sports and out-of-school sports/physical activities. Likewise, Wichstrøm and Wichstrøm [16] distinguished between the type of sport in terms of team sports (e.g., soccer, handball), individual sports (e.g., aerobics, horse riding), power sports (e.g., boxing, martial arts, weight lifting, gymnastics), endurance sports (e.g., running, skiing, speed skating), and technical sports (i.e., other sports), and they observed that the participation in team sports was positively associated with alcohol consumption and that participation in team sports and endurance sports was negatively associated with tobacco use. Kirckcaldy et al. [47] obtained the same results regarding regular involvement in endurance sports and the usage of cigarettes.

Pate et al. [48] indicated that male hockey and female soccer players (i.e., contact sports) were the most likely to report high levels of alcohol consumption, whereas cross-country athletes (i.e., non-contact sport) reported the lowest levels. Nevertheless, Peretti-Watel et al. [46] analyzed the differences between the regular practice in individual or a team sport, and found that both were correlated positively with the frequency of alcohol use. Although the results of the present study are in line with most of these results, they cannot be compared completely, because in this study, the type of sports was analyzed in terms of non-contact (i.e., low-contact) and contact (medium- and high-contact) sports. In this regard, some studies have also observed that athletes in contact sports had a greater likelihood of using substances than athletes in non-contact sports [49,50].

Regarding the action tendencies, it was found that athletes who showed more aggressiveness in their sport were more likely to smoke tobacco during the last 30 days and also to drink more alcohol throughout life that the less aggressive athletes. These results are consistent with prior literature that supports the associations between aggressiveness and alcohol and tobacco use among adolescents [27,51]. In this regard, it has been proposed that contact sport athletes, such as handball and basketball players, are more aggressive than non-contact sport athletes [52], which would also be in line with the higher alcohol and tobacco rates of consumption observed among the adolescents of this study participating in basketball and rugby.

While aggressive tendencies in sports were positively related with both tobacco and alcohol consumption, the other action tendencies did not show any significant relationship with tobacco use among adolescent athletes. Therefore, it could be proposed that reducing aggressive behavior in sports should be a priority, not only for the enhancement of the sport itself, but also to prevent alcohol and tobacco consumption, especially among adolescents who participate in medium- and high-contact sports.

Finally, the athletes who reported more submissiveness in their sport were less likely to have drunk alcohol during the last 12 months and 30 last days. This result is also consistent with studies that analyzed drinking behavior in other contexts. For instance, Franzen et al. [53] observed that drinking alcoholic beverages was negatively associated with submissive behavior. This could be explained on the basis of the notion that sports participation is a social activity where the contact with other peers may increase the risk of drinking [16]. According to the Big Five model, extroversion (i.e., the opposite of introversion) is negatively (and neuroticism positively) associated with submissiveness in children [25]. Higher levels of neuroticism may cause children to react in a shy and inhibited manner to stressful social situations [54]. Moreover, low extroversion levels may lead to less participation in social situations and to refuse to participate in social activities [54,55], whereas high extroversion is a significant predictor of alcohol consumption in adolescence [56].

Therefore, since submissiveness characterizes introverted behaviors [57], it could be proposed that athletes who behave submissively in their sports may also experience some kind of inhibition against spending too much time with their peers outside of the sport context and, therefore, to have less likelihood of participating in meetings where alcohol is present. In this regard, Nikel [25] indicates that aggressiveness and submissiveness have been considered opposite types of assertiveness reactions, but also as theoretical constructs completely independent of each other. According to the results of this study, a hypothetical continuum in the sport context might be connected to the alcohol consumption in adolescent athletes. However, since no studies examining the relationship between substance use among adolescent athletes and their action tendencies in sports were found in the scientific literature, only a hypothetical explanation can be proposed. Hence, more research is needed and the following limitations should be taken into consideration.

The first limitation is that the study only involved adolescent athletes from six sports. Second, although prior studies showed that the smoking frequency in adolescence assessed by self-report questionnaires was nearly identical to the frequency assessed by biological markers [58], alcohol and tobacco use were measured using a self-report questionnaire in a transversal manner. Therefore, biases inherent to self-reporting may have occurred. Regarding the questionnaire, it should be considered that at the age of 15 years, both tobacco smoking and alcohol drinking are in their development phase and, therefore, asking for the lifetime use could not have provided a valid measure for this age [27]. On the other hand, alcohol and tobacco consumption could also vary by the degree of involvement and the level of competition. In addition, variables that consider sexist behaviors that can occur in sports and during adolescence could be controlled for in future research. Another limitation of this study was not adding parental behavior as a variable. Since parents can drink and smoke, the children could be more likely to show this type of behavior. Since these variables were not considered in the design of the present study, future studies might analyze their effects. Finally, the study was designed to be cross-sectional and the results show no causal relationships. Likewise, the results can only be interpreted in the context of competitive sports, because adolescents who usually practice recreational sports could have different habits and behaviors. These limitations should be taken into consideration in the future.

## 5. Conclusions

The results of the present study indicated that age is an important risk factor for adolescent athletes when it comes to alcohol and tobacco use. Likewise, the type of sport and the adolescent action tendencies (i.e., aggressiveness and submissiveness) also showed a predictive value for the use of these substances in adolescent athletes. This is important because some practical implications can be derived from these results. Interventions to target a specific set of risk factors for alcohol and tobacco use could be implemented in schools and in sports clubs. At the age of 15 and 16, primary prevention programs and learning experiences that improve personal and social competence skills might have a beneficial effect on various psychological factors associated with reduced alcohol and tobacco use [59]. For instance, athletes should become involved in programs that consist of 15 class periods (about 45 min each) during the competitive season. These interventions should continue for two additional years.

## Figures and Tables

**Table 1:** Ordinal logistic regression analysis examining the impact of the type of sport (contact vs. non-contact) age, gender, personal and social responsibility, and action tendencies in life and in sport (aggressiveness, assertiveness, submissiveness) on the number of cigarettes smoked throughout life.

	Estimate	Std. Error	Wald	Sig. (*p*)	95% Confidence Interval
Age					
12 years old	−3.096	0.767	16.287	0.000	−4.600; −1.593
13 years old	−1.739	0.419	17.215	0.000	−2.560; −0.917
14 years old	−1.733	0.453	14.669	0.000	−2.620; −0.846
15 years old	−1.937	0.562	11.873	0.001	−3.039; −0.835
16 years old	1				
Type of Sport					
Non-contact	−0.925	0.346	7.154	0.007	−1.603; −0.247
Contact	1				

Note: Only significant variables are included in the table.

**Table 2:** Ordinal logistic regression analysis examining the impact of the type of sport (contact vs. non-contact) age, gender, personal and social responsibility, and action tendencies in life and in sport (aggressiveness, assertiveness, submissiveness) on the number of cigarettes smoked during the last 12 months.

	Estimate	Std. Error	Wald	Sig. (*p*)	95% Confidence Interval
Age					
12 years old	−3.708	1.053	12.406	0.000	−5.771; −1.645
13 years old	−2.257	0.495	20.768	0.000	−3.227; −1.286
14 years old	−2.096	0.511	16.796	0.000	−3.098; −1.093
15 years old	−2.494	0.679	13.507	0.000	−3.824; −1.164
16 years old	1				
Type of Sport					
Non-contact	−1.239	.407	9.280	0.002	−2.036; −0.442
Contact	1				

Note: Only significant variables are included in the table.

**Table 3:** Ordinal logistic regression analysis examining the impact of the type of sport (contact vs. non-contact) age, gender, personal and social responsibility, and action tendencies in life and in sport (aggressiveness, assertiveness, submissiveness) on the number of cigarettes smoked during the last 30 days.

	Estimate	Std. Error	Wald	Sig. (*p*)	95% Confidence Interval
Age					
12 years old	−2.756	1.089	6.407	0.011	−4.889; −0.622
13 years old	−2.328	0.705	10.916	0.001	−3.709; −0.947
14 years old	−3.392	1.084	9.787	0.002	−5.518; −1.267
15 years old	−2.918	1.095	7.102	0.008	−5.065; −0.772
16 years old	1			.	.
Type of Sport					
Non-contact	−1.573	0.598	6.919	0.009	−2.746; −0.401
Contact	1				
Aggressiveness in Sport	0.127	0.054	5.475	0.019	0.021; 0.233

Note: Only significant variables are included in the table.

**Table 4:** Ordinal logistic regression analysis examining the impact of the type of sport (contact vs. non-contact) age, gender, personal and social responsibility, and action tendencies in life and in sport (aggressiveness, assertiveness, submissiveness) on the number of alcoholic beverages drunk throughout life.

	Estimate	Std. Error	Wald	Sig. (*p*)	95% Confidence Interval
Age					
12 years old	−2.646	0.525	25.358	0.000	−3.676; −1.616
13 years old	−2.322	0.405	32.823	0.000	−3.116; −1.528
14 years old	−0.823	0.346	5.669	0.017	−1.501; −0.146
15 years old	−0.491	0.385	1.629	0.202	−1.246; 0.263
16 years old	1				
Type of Sport					
Non-contact	−0.590	0.268	4.829	0.028	−1.116; −0.064
Contact	1				
Aggressiveness in sport	0.109	0.027	16.126	0.000	0.056; 0.162

Note: Only significant variables are included in the table.

**Table 5:** Ordinal logistic regression analysis examining the impact of the type of sport (contact vs. non-contact) age, gender, personal and social responsibility, and action tendencies in life and in sport (aggressiveness, assertiveness, submissiveness) on the number of alcoholic beverages drunk during the last 12 months.

	Estimate	Std. Error	Wald	Sig. (*p*)	95% Confidence Interval
Age					
12 years old	−2.775	0.530	27.448	0.000	−3.783; −1.720
13 years old	−2.627	0.429	37.459	0.000	−3.509; −1.840
14 years old	−1.051	0.343	9.377	0.001	−1.815; −0.468
15 years old	−0.429	0.371	1.337	0.176	−1.222; 0.224
16 years old	1				
Submissiveness in Sport	−0.201	0.044	20.549	0.000	−0.288; 0.114

Note: Only significant variables are included in the table.

**Table 6:** Ordinal logistic regression analysis examining the impact of the type of sport (contact vs. non-contact) age, gender, personal and social responsibility, and action tendencies in life and in sport (aggressiveness, assertiveness, submissiveness) on the number of alcoholic beverages drunk during the last 30 days.

	Estimate	Std. Error	Wald	Sig. (*p*)	95% Confidence Interval
Age					
12 years old	−2.266	0.527	18.465	0.000	−3.299; −1.232
13 years old	−2.026	0.415	23.860	0.000	−2.839; −1.213
14 years old	−1.076	0.369	8.488	0.004	−1.800; −0.352
15 years old	−0.0374	0.388	0.928	0.335	−1.135; 0.387
16 years old	1				
Submissiveness in Sport	−0.167	0.046	13.358	0.000	−0.257; −0.078

Note: Only significant variables are included in the table.

## Data Availability

The research data can be made available. Any further inquiries can be directed to the authors.
